# WHAT CAN HISTORY DO FOR BIOETHICS?

**DOI:** 10.1111/j.1467-8519.2011.01933.x

**Published:** 2011-12-13

**Authors:** Duncan Wilson

**Keywords:** history, bioethics, empirical turn, pluralism

## Abstract

This article details the relationship between history and bioethics. I argue that historians' reluctance to engage with bioethics rests on a misreading of the field as solely reducible to applied ethics, and overlooks previous enthusiasm for historical perspectives. I claim that seeing bioethics as its practitioners see it – as an interdisciplinary meeting ground – should encourage historians to collaborate in greater numbers. I conclude by outlining how bioethics might benefit from new histories of the field, and how historians can lend a fresh perspective to bioethical debates.

## INTRODUCTION: BIOETHICS AND THE RELUCTANT HISTORIAN

Of all the questions bioethicists have tackled in recent decades, few have proved as enduring as: what exactly is bioethics, and who qualifies as a bioethicist? Bioethics was represented as a collaborative enterprise when it emerged in the United States during the late-1960s – largely in order to differentiate it from medical self-regulation, which was heavily criticized in discussion of human experiments and new biomedical technologies. Participants included philosophers, theologians, lawyers, sociologists, clinicians and biomedical scientists; and Warren Reich's *Encyclopedia of Bioethics* defined it as ‘an area of interdisciplinary studies’ concerned with ‘the systematic study of human conduct in the area of the life sciences and health care’.^1^ Yet by the mid-1970s, as Daniel Callahan notes, analytically trained philosophers became ‘the dominant force in the field’.^2^ Keen to distance themselves from the mid-20th-century interest in logical positivism and meta-ethics, they viewed bioethics as a vehicle for the practical application of consequentialist, utilitarian and rights-based approaches to ethics.^3^ For the ethicist Danner Clouser, chair of bioethics at Penn State Medical School, bioethics involved application of ‘old’ ethical principles to medical dilemmas, and the philosopher was critical in clarifying the major issues, isolating concepts and helping avoid logical inconsistencies.^4^

The view that bioethics was a form of applied ethics was captured in Tom Beauchamp and James Childress's 1979 book *Principles of Biomedical Ethics*. Beauchamp and Childress claimed that ethical problems could be resolved through the application of four principles: autonomy, non-maleficence, beneficence and justice.^5^ Crucially, they differentiated these normative principles, which were concerned with determining what ought to be the case, from the empirical work done by social scientists, which they believed simply described how things were and was ‘secondary’ to bioethics (it is perhaps no coincidence that Beauchamp was an expert on David Hume, who famously argued that one could not derive a moral ‘ought’ from a factual ‘is’).^6^ These arguments certainly appeared convincing: philosophers, or what Peter Singer called ‘moral experts’, were an influential presence on federal bodies like the National Commission for Protection of Human Subjects in Biomedical Research and were largely responsible for its 1979 recommendations, known as the *Belmont Report*, which argued that research should conform to the principles of respect for persons, beneficence and justice.^7^

But this emphasis on philosophy sidelined those sociologists and anthropologists who had also studied ethical issues in healthcare during the 1970s. Unsurprisingly, some criticized the shortcomings of ‘mainstream’ bioethics during the 1980s. Renee Fox and Judith Swazey, for instance, argued that it was marked by a worrying tendency to ‘distance and abstract itself from the human settings in which ethical questions are embedded and experienced’.^8^ Bioethics was beset by a ‘cultural myopia’,^9^ they claimed, and:

if it is an indicator of the general state of American ideas, values, and beliefs … then there is every reason to be worried about who we are, what we have become, what we know, and where we are going in a greatly changed society and world.^10^

Notably though, Fox and Swazey admitted that the ‘limited participation’ of anthropologists and sociologists was ‘caused as much by the prevailing intellectual orientations and weltanschauug of present-day social science as by the framework of bioethics’.^11^ Trained to analyse situations rather than speculate about how they ought to be, social scientists shied away from making any contribution and simply critiqued bioethics – acting, to a recent review, like ‘the team member who does nothing to help but only criticizes team performance’.^12^ Philosophers took offence at this negative characterization of their work; and in 1990 Fox admitted that relations between bioethics and the social sciences remained ‘tentative, distant and susceptible to strain’.^13^

Relations thawed in the 1990s, however, as social scientists began to outline how bioethics might benefit from adopting sociological and ethnographic perspectives.^14^ To the sociologist Charles Bosk, their main contribution was ‘the provision of context, the gentle insistence that principles are attached to persons, and the constant reminder that those persons have interests, a history and a culture’.^15^ Bosk and others argued that a more ‘bottom up’ approach, based on a dense knowledge of particular social settings, could help connect bioethics to the actual expectations of doctors or patients, who regularly displayed preferences, values and forms of reasoning different from those prioritized in bioethical texts.^16^ Some even argued this work transcended the division between normative and descriptive ethics, by demonstrating how morality is embedded in, and produced through, the very practices, objects and relationships that social scientists investigate.^17^ These arguments found a receptive audience; and as social scientists published in bioethical journals and helped determine public policy, many talked of an ‘empirical turn’ in bioethics.^18^ Now, sociologists, anthropologists and economists, amongst others, confidently describe bioethics as a ‘dynamic, changing, multi-sited field’ where many participants ‘claim the title of bioethicists’.^19^

But one professional group remains conspicuously absent from this ‘multi-sited field’. Historians of science, technology and medicine have documented the moral issues associated with specific medical and biological practices – e.g. human experiments, vivisection, compulsory vaccination, abortion and organ transplantation – yet few have sought to engage with bioethics. The only real collaboration came in the late-1970s, when several American historians discussed the historical background to contemporary ethical issues in bioethical journals and institutions. These included Gerald Geison, Martin Pernick, David Rothman, Allan Brandt and James Jones, whose 1981 book on the Tuskegee syphilis study, *Bad Blood*, was written partly at Harvard University's Interfaculty Program in Bioethics and has been described by Arthur Caplan as ‘the single most important book ever written in bioethics’.^20^ Summarizing a conference on ‘Ethics and Sensibility in the History of Science and Medicine’, held at the Hastings Center in 1977, Karen Lebacqz hoped these historians might assist bioethicists ‘gain perspective on current debates’.^21^ While figures like David Rothman cautioned that history provided ‘no firm answers’, they claimed it could at least help bioethics ‘ask the right questions’ by rooting moral positions in specific social, cultural and historical contexts.^22^

But this association was brief. By the 1980s, many historians in the United States and elsewhere emulated sociologists and anthropologists by distancing themselves from bioethics. Although they continued to historicize ethical issues in medicine and science, they no longer wrote in bioethics journals or spoke at ethics conferences. If they considered bioethics at all, historians instead treated it as an object of study and criticized bioethicists for what Roger Cooter called a ‘shallowness (or absence) of socio-economic and political understanding’.^23^ Charles Rosenberg, to take another example, rebuked bioethicists for consistently failing to acknowledge how:

the ways in which the moral values that suffuse medicine are historically constructed and situationally negotiated, like every other aspect of culture, and not simply derived from the formal modes of analysis that have historically characterized theology and moral philosophy.^24^

Historians claimed this tendency ‘to de-contextualize the ethical in medicine’ was crucial in helping bioethicists acquire professional and public authority, allowing them to present their arguments as ‘usable in a value-free manner’.^25^

In documenting how bioethics gained prestige, historians also debunked participant accounts they claimed misleadingly portrayed bioethicists as radical critics of medicine.^26^ Undermining the links that are often presumed to exist between bioethics and the civil rights movement, Tina Stevens argued that ‘bioethical impulses found their way into enduring social institutions not because they represented the social challenges of the 1960s but because they successfully diffused those challenges’.^27^ Stevens concluded that bioethics gained influence because it helped legitimate research and clinical practice: avoiding fundamental questions about medical power and formulating guidelines ‘for the use of procedures and technologies that it largely accepted as inevitable’.^28^ Rosenberg, too, detailed how ‘bioethics has taken up residence in the belly of the medical whale’ and is ‘no longer, if it ever was, a free-floating, oppositional and reform movement’.^29^ For Jonathan Imber, meanwhile, bioethicists were little more than ‘the public relations division of modern medicine’.^30^

These accounts embody what Georg Simmel called ‘negative solidarity’: historians agree on what is wrong with bioethics, but show little inclination to convert their skepticism into a positive agenda for change.^31^ Any hopes for a constructive dialogue were further undermined by the fact that bioethicists reacted badly to being so roundly criticized. Albert Jonsen accused historians of trying to bully ‘the new kid on the block’ and rejected the characterization of bioethicists as ‘pusillanimous opportunists [and] subservient apologists for the medical establishment’.^32^ And Arthur Caplan dismissed Cooter's claim that bioethics was intellectually conservative and ‘destined for a short lifespan’ as ‘amazingly flawed … intellectual tripe’.^33^ This was reminiscent of the ‘tiresome back and forth’ that marked exchanges between bioethicists and social scientists during the 1980s.^34^ Historians like Cooter admitted ‘no real desire to contribute’, while bioethicists appeared irritated by the regular criticism.^35^ Neither side regretted that history was outside bioethics and, despite the empirical turn, there seemed little chance of a rapprochement.

## EXPLAINING RELUCTANCE: A MISREADING OF BIOETHICS?

There are several possible reasons for historians' refusal to contribute to bioethics. Firstly, as Jonsen and Caplan hinted, they may simply resent being sidelined by the ‘new kid on the block’. As Mark Jackson notes, while ‘historians struggle to elaborate precisely what the study of history (or indeed other humanities) can offer present and future clinicians’, bioethicists have had no qualms asserting their relevance.^36^ Consequently, in Britain and the United States, bioethics has largely superceded history as a means of introducing students and doctors to humanist values in medical practice, with its graduate programmes attracting more students and money than comparable degrees in the history of medicine and science (this is more pronounced in Britain than the United States, where some universities have joint departments of history and bioethics, and historians are employed in bioethics departments).

Secondly, many historians have long argued the discipline will be ‘dumbed down’ if it engages too much with contemporary issues.^37^ This hinges on the same ‘is-ought’ distinction that led many sociologists and anthropologists to distance themselves from bioethics in the 1980s. Historians believe their task is to recover the lives of past individuals, cultures or social groups, and are uneasy about extrapolating from this to state how things ought to be in the present. Gerald Geison hinted as much in 1978, when he claimed that adopting an ‘explicitly normative stance’ ran the risk of doing ‘major violence to historical sensibilities’.^38^ This reluctance has been deepened by the postmodern view that history is not the objective gathering of ‘facts’ about the past, but is rather a narrative construct that reflects the ideological bias of the author.^39^ To Keith Jenkins, the belief that history writing results in the production of highly subjective narratives impels historians to admit that ‘no judgement is definitive’.^40^ It follows from this, therefore, that ‘there never will be any entailed connection between history and ethics’, and historians should shy away from engaging with practical affairs.^41^

While these are both plausible explanations, I believe the major factor behind historians' reluctance lies in a misreading of bioethics. As is clear from the preceding section, historians equate bioethics with the ‘applied ethics’ model promoted during the late 1970s: operating through the application of normative principles, without considering social, cultural or historical factors. Roger Cooter, for one, claimed that bioethics ‘hardly stirred historians’ because of its failure to ‘consider the socio-economic and political possibilities for and constraints upon asking the “right” questions and arriving at the “right” ethical conclusions’.^42^ Cooter believes the fundamental incongruity between historical and bioethical methodologies renders historians ‘speechless in contempt, silent for political reasons, dumb for fear of offending’.^43^ What is more, he presents history as fundamentally inimical to this ‘socially transcendent’ enterprise:

Authority in medical ethics is meant to emerge from the rigour of its socially-detached philosophical logic; for medical ethics to be historical would threaten this primary means to authority. History is therefore subversive to the essential purpose of medical ethics; its only conceivable role is the ludicrously Whiggish one of revealing ‘improvements’ in morals over time.^44^

But this is more a caricature than an accurate portrayal. To quote de Vries, Turner et al., it falls into the trap of ‘identifying bioethics as if it were a monolithic entity, with a single perspective and mode of inquiry, reinforced by a cadre of leaders whose position and expertise are unchallenged’.^45^ While the ‘applied ethics’ model was a significant part of American bioethics during the late-1970s and 1980s, historians have overlooked that bioethicists endorsed alternative approaches, which involved greater emphasis on social, cultural and historical perspectives. This enthusiasm did not come from some marginal fringe, moreover, but came from principal figures in the history of bioethics. In 1982, for instance, Daniel Callahan, a founding member of the Hastings Center, claimed no one field ‘had a special claim on the fundamental methodology’ in bioethics, and reported growing dissatisfaction with its ‘indifference to history, social context, and cultural analysis’.^46^

Callahan was not alone in bemoaning the relative absence of social and historical work in bioethics. During the late-1970s, Alasdair MacIntyre criticized bioethicists for refusing to acknowledge that different moral positions on a particular issue, such as abortion, were the product of specific social and historical traditions, and could not be fully comprehended without recourse to these broader factors.^47^ Although his association with bioethics was brief, and while he never considered himself a bioethicist, MacIntyre was no outsider: he acted as a consultant for the National Commission for Protection of Human Subjects and, like Callahan, published in the *Hastings Center Report*. In the same period, the philosopher Laurence B. McCullough also complained that ‘present undertakings in biomedical ethics lack the sort of firm foundation that sustained historical reflection can provide’.^48^ McCullough was not advocating approaches that historians dismiss as ‘Whiggish’, such as searching for past instances of contemporary concerns, or seeking to position bioethics as evidence of moral progress.^49^ Rather, he called for a systematic effort to root ethical codes in their correct historical context: determining their broader influences and ascertaining why certain standards changed or endured over time. This, he concluded, would allow bioethicists to better appreciate ‘the new claims and conditions that are placed on [the doctor–patient] relationship from outside and which may make it impossible for some of the older dimensions to exist’.^50^

In Britain, where bioethics emerged in the 1980s, there was also no clear consensus in favour of applied ethics or its emphasis on individual autonomy. While Rannan Gillon advocated principalism in the *British Medical Journal*, the philosopher Robin Downie claimed that ethical questions also involved ‘politics and power’, and argued that prioritizing individual autonomy may not sit well in countries with a strong welfare state.^51^ To the academic lawyer Ian Kennedy, whose 1980 BBC Reith Lectures called for external scrutiny of medicine and the teaching of medical ethics by ‘outsiders’, bioethics was a diverse field that involved ‘ethics and law, together with sprinklings of philosophy, sociology and politics’.^52^ Kennedy's promotion of bioethics drew on philosophy, legal cases, theorists like Michel Foucault, radical critics like Ivan Illich, and medical reformers like Thomas McKeown and Muir Gray. Some of these figures, such as Foucault, Illich and McKeown, also influenced medical historians; and Kennedy's calls for greater patient involvement in medical decisions echoed their demands for a bottom up ‘patient's history of medicine’.^53^

**Figure 1:**
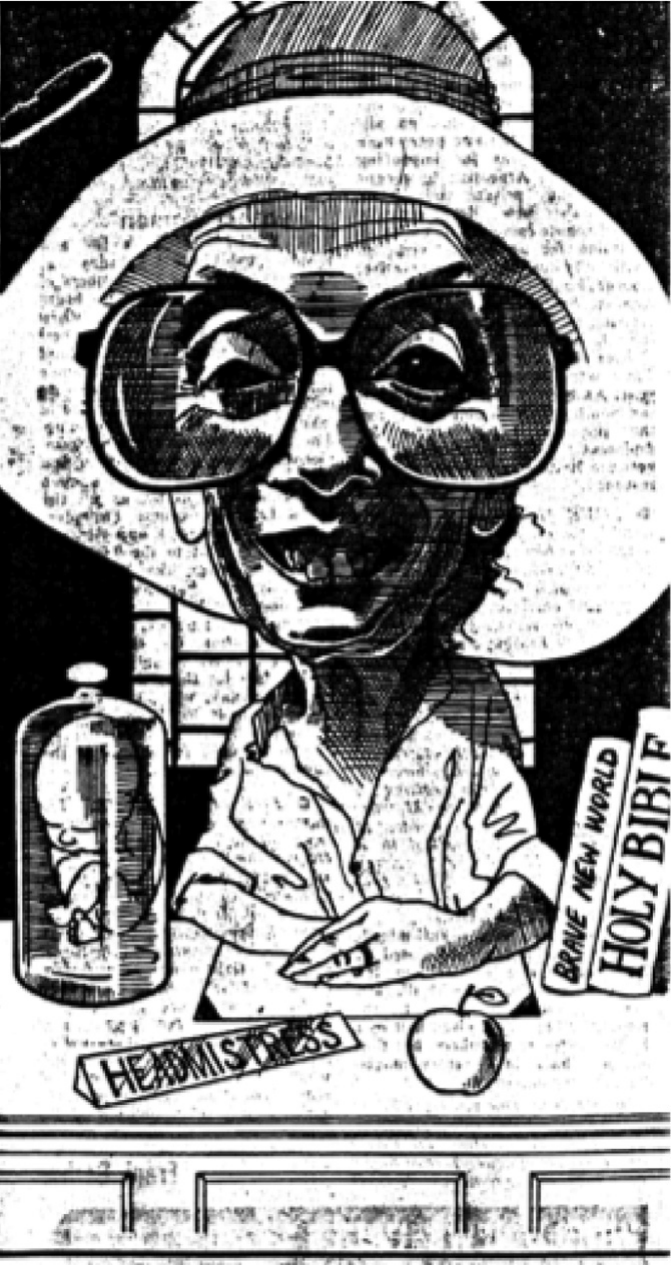
Newspaper caricature of Mary Warnock. Reproduced courtesy of Joe Cummings.^59^

The philosopher Mary Warnock (see figure 1), who chaired a government inquiry into human fertilization and embryology between 1982 and 1984, also rejected claims that bioethics was simply a vehicle for philosophy. Responding to Peter Singer's endorsement of ‘moral experts’, Warnock declared that ‘no-one is prepared to defer to judgments made on the basis of superior ability in philosophy’.^54^ To Warnock, ‘there was no such thing as a moral expert’, and bioethics should function as a form of ‘corporate decision-making’ for various professions and interest groups.^55^ This view clearly proved influential. Warnock's support for a British bioethics council influenced the 1991 formation of the Nuffield Council on Bioethics, where philosophers served alongside lawyers, clinicians, biomedical scientists, sociologists, lawyers, theologians, businessmen, journalists and others.^56^ To Onora O'Neill, a former president of the Nuffield Council, this arrangement ensures that bioethics ‘is not a discipline’, but instead provides ‘a meeting ground for a number of disciplines, discourses and organizations concerned with ethical, legal and social questions raised by advances in medicine, science and technology’.^57^

Appeals for historical input intensified during the empirical turn, as social scientists claimed history was essential for charting ‘the transformation of certain practices from being “ethical concerns” to “mundane practices” and back again’.^58^ Yet despite this and earlier appeals, historians remained on the sidelines and complained that bioethics was marked by ‘retention of narrow analytical notions’ and the ‘exclusion of alternative perspectives’.^60^ It was little wonder, then, that bioethicists believed those historians who steadfastly refused to contribute had ‘only a superficial acquaintance’ with the field: mistakenly portraying it as one approach, rather than as ‘a discourse where many people discuss, argue, and attempt to resolve perplexities into decisions and policies’.^61^

## THE WAY FORWARD: HISTORY OF BIOETHICS AND HISTORY IN BIOETHICS

If historians are to overcome their longstanding skepticism, they should view bioethics as its practitioners do – as an interdisciplinary ‘meeting ground’ where they can make a decisive contribution. To achieve this, they need to undertake more contextualized histories of bioethics: charting how its outlook, methods and composition varied according to professional agendas and broader social concerns in different times and places. This new approach is evident in contributions to Robert Baker and Laurence McCullough's 2009 *World History of Medical Ethics*, and in detailed histories of bioethics in Britain, Colombia and Singapore, which encouragingly define their object of study as ‘an assemblage of knowledge, expertise and techniques’.^62^

This new work promises to move us beyond the rather simplistic assumption that bioethics was simply a product of civil rights politics, or the inherently problematic nature of new technologies. In Britain, for example, despite controversies over Thalidomide, organ transplants and the definition of death during the 1960s and 1970s, the lawyers, philosophers and theologians who discussed medical ethics did not critique medicine or seek to involve themselves in its regulation. As Alastair Campbell stated in the *Journal of Medical Ethics*, they believed ‘the final decisions remain medical ones’ and their role was to ‘help doctors make more informed decisions’.^63^ In 1978 the *British Medical Journal* contrasted this with the ‘American trend’ of bioethics, where philosophers, theologians and lawyers acted ‘as society's conscience in matters once left to the medical profession’.^64^‘Bioethics’ did not emerge as a term or approach here until the early 1980s, when figures like Ian Kennedy and Mary Warnock publicly argued that ‘increasingly, and rightly, people who are not experts expect, as of right, to determine what is or is not a tolerable society to live in’.^65^ These demands were influential, in no small part, because they resonated with the Conservative government's desire to reform professions – exposing them to outside scrutiny in order to make them more efficient and publicly accountable.^66^ Ministers recruited growing numbers of ‘outsiders’ to regulatory committees, and supported Warnock and Kennedy's demands for a national ethics council (although they were wary of linking it directly to Whitehall).

These histories also promise to overcome the binary view of bioethics as either a radical critique of medicine, or its apologetic ‘public relations division’. Although Warnock and Kennedy called for an end to self-regulation, they promised this would benefit doctors by aligning medicine to changing political and public expectations. Kennedy stressed that outsiders were ‘trying to help’, and Warnock claimed oversight was ‘essential if we are to continue, as we must, to push back the frontiers of science’.^67^ Many doctors, too, acknowledged that ‘the era of paternalism is past’ and that bioethics was necessary ‘to follow the rhetoric of the present government’.^68^ This helps us to see figures like Kennedy and Warnock, and bioethics in general, as vital intermediaries between politicians, the public and the medical profession: whose criticism was designed to reconcile doctors to a changing political landscape.

History also complements work in the empirical turn by problematizing the dichotomy between the empirical and normative, and between science and ethics. For example, the Warnock committee's recommendations for embryo experiments hinged on biological theories of development. Aware that her committee was split between supporters and opponents of embryo experiments, Warnock recognized there could be no ‘correct’ answer. Her task instead was to seek ‘something practical, regretted no doubt by some as too lax, by others as too strict, but something to which, whatever their reservations, everyone would be prepared to consent’.^69^ The developmental biologist Anne McLaren, who Warnock describes as ‘indispensible’, informed fellow committee members that around fourteen days after fertilization the cells of the rudimentary embryo condense to form the ‘primitive streak’, which differentiates into the antecedents of the spinal cord and nervous system.^70^ McLaren claimed the primitive streak offered an ethical cut-off for experiments, as it marked the first point where there was any chance of an embryo experiencing pain. Another factor making the primitive streak a good cut-off was the fact that it also marked the last point where an embryo could divide to form identical twins, which McLaren believed made it hard for opponents to frame the stages beforehand as a potential individual.

Taking the broadly utilitarian view that embryo experiments were vital to understanding development and treating childhood diseases, the majority of the committee agreed to adopt fourteen days as the limit for research. Crucially, their report portrayed the primitive streak as a significant biological *and* ontological landmark: where ‘a loose ball of cells’ acquired the distinguishing features of the ‘embryo proper’.^71^ Warnock even claimed the primitive streak settled philosophical questions of when a human individual could be said to begin. Writing in the first edition of *Bioethics*, she asserted that:

Up to the [primitive streak] it is difficult to think of the embryo as an individual, because it might still become two individuals. None of the criteria that apply to me, or Tom or Dick or Harry, and distinguish us from the others, are satisfied by the embryo at this early stage. The collection of cells, though loosely strung together, is hardly yet one thing, nor is it several … But from the fourteenth or fifteenth day onwards, there is no doubt that it is Tom or Dick or Harry that is developing.^72^

History thus helps us appreciate how ethics is not derived from abstract philosophical principles, but is produced by a dynamic engagement between scientific theories, moral frameworks such as utilitarianism, and the rhetoric of individuals like Warnock. This can help bioethicists reflect on the value and meaning of their work: on the strategies they employ to designate certain issues as ‘bioethical’, and how this is ‘boundary work’ is socially embedded.^73^ As Jonsen concedes, even if they disagree with the findings, history can make bioethicists ‘more attentive to what we are, to what we do, and how we may be perceived’.^74^

In turn, a better understanding of bioethics should encourage historians to outline how it can benefit from their own expertise – moving us from ‘history *of*’ bioethics to ‘history *in*’ bioethics.^75^ They should promote history not just in the usual outlets, but also in bioethical journals and conferences where, after all, philosophers, lawyers, sociologists and others utilize and endorse their own methods. The advantages of this move are twofold: lending balance and a vital perspective to ethical debates, and giving historians the chance to engage with practical affairs when universities and research councils increasingly prioritize ‘impact’. There are promising signs that such collaboration is increasing. Interdisciplinary funding programmes in ‘medical humanities’ encourage historians to work alongside colleagues in other fields; historians of science, technology and medicine, such as David Edgerton, now advise bodies like the Nuffield Council on Bioethics; and new interdisciplinary journals like *Biosocieties* or *Medicine Studies* provide historians with the chance to assert the value of history to current debates on eugenics, regenerative medicine and drug development.^76^

As this burgeoning collaboration demonstrates, integrating history into bioethics does not mean compromising historical methodologies or abandoning earlier critiques of bioethics. Rather, it involves making them available as an intellectual resource. Charles Rosenberg, for example, argues that history's core principles of ‘context, context, and context’ can help bioethicists understand the ‘time and place-specific structure of choices as perceived by particular actors’.^77^ History complements anthropology and sociology, he claims, by reminding bioethicists there ‘can be no decontextualized understanding of bioethical dilemmas’.^78^

Neither does it mean a rejection of the postmodern view of history, and a return to a naïve belief in historical objectivity. Historians should not be discouraged by claims that history is the subjective interpretation of the author. Theirs is not the only discipline whose methods and objectivity have been questioned in this fashion. Historians have done likewise for the sciences, anthropologists have asked troubling questions of ethnography, and philosophers like George Agich have stated that clinical ethics is ‘a primarily interpretive practice’ that is shaped ‘by the actions, perceptions and judgments of the consultant and other individuals involved in a consultation case’.^79^ All these disciplines contribute to bioethics, so why not history? As long as we have confidence in their professional integrity, there is no reason why disagreement between historians need be any more troubling than between scientists, sociologists or philosophers.

Indeed, Hayden White claims that pluralist views of history need not distance the field from ethics. Rather, they can help foster ‘greater tolerance and efforts to understand the other’ by demonstrating the existence of many different moral positions on a particular issue – which are neither ‘true’ nor ‘false’, but cannot be comprehended without recourse to the political, social and cultural milieu in which they arise.^80^ History here provides a way of broadening ethical debates beyond a focus on the pros and cons of moral positions. It helps to link these positions to social contexts, and allows us to ascertain their origins and pertinence.^81^ By rooting particular standpoints in different social, political and religious traditions, historians can help reconcile bioethics to the challenges posed by pluralist societies where, as Warnock recognized, ‘there do not exist uniform or universal moral sentiments’.^82^

In doing so, history lends an important perspective on resistance to innovations. In a recent discussion of the hand-knitters who destroyed industrial weaving machines in the early-19th-century known as ‘Luddites’, David Edgerton claims that rejecting innovation has long been a necessary part of scientific and social progress. ‘Most people said to be Luddites today’, he argues, ‘are not against progress in science or technology in general, but against particular manifestations in particular contexts’.^83^ Edgerton contends that discussion can only be ‘raised above its current, depressingly low level’ once we seriously attend to the particular contexts that underpin resistance. Like other fields in the empirical turn, history here provides bioethics with a chance to engage seriously with popular opposition – rather than simply dismissing it as ill-informed ‘prejudices’.^84^ This will doubtless broaden our understanding of the moral positions taken on issues like genetic screening and post-implantation diagnosis, where watchdogs and disability groups frequently invoke history in their references to a ‘new eugenics’.^85^

But history does more than just complement other approaches in the empirical turn. It brings its own specific benefits and promises to expand the focus of bioethics.^86^ Historical work can help shift bioethics away from its focus on new and emerging technologies, which may not impact on the day-to-day lives of patients, to a broader consideration of the role politics plays in shaping medical services. Many historians are well placed to comment on debates regarding proposed reforms of the National Health Service, which currently lack any discussion of what impacts previous changes had on patient care. As John Pickstone notes, when hardly anyone remembers what the NHS was like in 1995, let alone 1955, and when debate is marred by obvious sectional interests, history is vital for restoring narrative, balance and a consideration of motives and their effects.^87^ By looking to the past on this and other issues, bioethics might find valuable ways of preparing for future change.

## Footnotes

1 W. Reich. 1978. Introduction. In *The Encyclopedia of Bioethics*. W. Reich, ed. New York: Macmillan: xv-xxii, on xix. Members of different disciplines had begun to discuss the ethical aspects of science and medicine by the late-1960s, but the term ‘bioethics’ did not emerge until 1970. It was first coined by the biochemist Van Rensselaer Potter, who used it to describe an ethics derived from biomedicine. Months later, Andre Helleger and Sarget Shriver used it in its now more familiar sense – i.e. to describe the ethical examination of medicine and biology – when they opened an Institute for the Study of Human Reproduction and Bioethics at Georgetown University, a private Jesuit college in Washington DC. See A Jonsen. 1998. *The Birth of Bioethics*. New York and Oxford: Oxford University Press.

2 D. Callahan. 1982. At the Center: From ‘Wisdom’ to ‘Smarts’. *Hastings Cent Rep* 1982; 12 no. 3: 4.

3 R. Fox & J. Swazey. 2008. *Observing Bioethics*. Oxford: Oxford University Press.

4 K.D. Clouser. 1978. Bioethics. In *Encyclopedia of Bioethics*. W. Reich, ed. New York: Macmillan: 124–125.

5 T. Beauchamp & J. Childress. 1979. *Principles of Biomedical Ethics*. First edn: New York & Oxford: Oxford University Press.

6 Ibid: 9.

7 Fox et al. *op. cit*. note 3, pp. 128–145. P. Singer. Moral Experts. *Analysis* 1972; 32: 115–117.

8 R. Fox & J. Swazey. Medical Morality is not Bioethics – Medical Ethics in China and the United States. *Perspect Biol Med* 1984; 27: 336–360, p. 356.

9 Ibid: 337.

10 Ibid: 360.

11 Ibid: 350.

12 R. de Vries, L. Turner, K. Orfali & C. Bosk. 2007. Social Science and Bioethics: The Way Forward. In *The View from Here: Bioethics and the Social Sciences*. R. de Vries, L. Turner, K. Orfali and C. Bosk, ed. Oxford: Blackwell Publishing: 1–12, p. 12.

13 R. Fox. 1990. The Evolution of American Bioethics: A Sociological Perspective. In *Social Sciences Perspectives on Medical Ethics*. G. Weisz, ed. Amsterdam: Kluwer: 201–217, on p. 213. For a dismissal of these critiques, see S. Gorowitz, ‘Baiting Bioethics’. *Ethics* 1986; 96: 356–374.

14 De Vries et al., *op cit*. note 12, p. 12.

15 C. Bosk. Professional Ethicist Available: Logical, Secular, Friendly. *Daedalus* 1999; 128: 47–69, p. 63.

16 A. Hedgecoe. Critical Bioethics: Beyond the Social Science Critique of Applied Ethics. *Bioethics* 2004; 18: 120–143; J. Lopez. How Sociology can save Bioethics … Maybe. *Sociol Health Ill* 2004; 26: 875–96; R. Zussman. The Contributions of Sociology to Medical Ethics. *Hastings Cent Rep* 2000; 30: 7–11; A. Kleinman. Moral Experience and Ethical Reflection: Can Ethnography Reconcile Them? A Quandry for the ‘New Bioethics’. *Daedalus* 1999; 128: 69–99; R. de Vries & P. Conrad. 1998. Why Bioethics Needs Sociology. In *Bioethics and Society: Constructing the Ethical Enterprise*. R. de Vries & J. Subedi, eds. New Jersey: Prentice-Hall: 233–258.

17 E. Haimes. What can the Social Sciences Contribute to the Study of Ethics? Theoretical, Empirical and Substantive Considerations. *Bioethics* 2002; 16: 89–113.

18 P. Borry, P. Schotsmans & K. Dierickx. The Birth of the Empirical Turn in Bioethics. *Bioethics* 2005; 19: 49–71.

19 De Vries et al., *op cit.* note 12, pp. 2–3.

20 A. Caplan. Bad Blood: The Tuskegee Syphilis Experiment. *Biosocieties* 2007; 2: 275–276; J.H. Jones. 1981. *Bad Blood: The Tuskegee Syphilis Experiment*. London: Free Press: 1–2. See also A.M. Brandt. Racism and Research: The Case of the Tuskegee Syphilis Study. *Hastings Cent Rep* 1978; 8, no. 6: 21–29; G.L. Geison. Pasteur's Work on Rabies: Reexamining the Ethical Issues. *Hastings Cent Rep* 1978; 8, no. 2: 26–33.

21 K. Lebacqz. At the Institute: Historians Discuss Ethical Issues of the Past. *Hastings Cent Rep* 1977; 7, no. 2: 3.

22 D. Rothman. Behavior Modification in Total Institutions. *Hastings Cent Rep* 1975; 5, no. 1: 17–24, p. 24.

23 R. Cooter. The Resistable Rise of Medical Ethics. *Soc Hist Med* 1995; 8: 257–270, p. 259.

24 C. Rosenberg. Meanings, Policies, and Medicine: On the Bioethical Enterprise and History. *Daedalus* 1999; 128: 27–46, pp. 36–37.

25 R. Cooter. 2000. The Ethical Body. In *Medicine in the Twentieth Century*. R. Cooter & J.V. Pickstone, eds. Amsterdam: Harwood Academic: 451–467, p. 453.

26 For examples of these ‘origin myths’ see, Jonsen, *op. cit.* note 1; S. Toulmin. How Medicine Saved the Life of Ethics. *Perspect Biol Med* 1982; 25: 736–750.

27 T. Stevens. 2000. *Bioethics in America: Origins and Cultural Politics*. Baltimore and London: Johns Hopkins University Press: xii.

28 Ibid: 158.

29 Rosenberg, *op. cit.* note 21, p. 38.

30 J.B. Imber. 1998. Medical Publicity Before Bioethics: Nineteenth Century Illustrations of Twentieth Century Dilemmas. In, *Bioethics and Society: Constructing the Ethical Enterprise*. R. de Vries & J. Subedi, eds. New Jersey: Prentice Hall: 16–38, p. 33.

31 On ‘negative solidarity’ toward bioethics in the social sciences, see de Vries et al., *op. cit.* note 12, p. 1.

32 A. Jonsen. Beating Up Bioethics. *Hastings Cen Rep* 2002; 32: 4–6, o p. 4.

33 R. Cooter. Historical Keywords: Bioethics. *Lancet* 2004; 364: 1749; A. Caplan. Reports of Bioethics’ Demise are Premature. *Lancet* 2005; 365: 654–655, p. 644.

34 De Vries et al., *op. cit.* note 12, p. 12.

35 Cooter, *op. cit.* note 20, p. 259.

36 M. Jackson. Back to the Future: History and Humanism in Medical Education. *J Med Educ* 2002; 36: 506–507. See also N. Emmerich. Literature, History and the Humanization of Bioethics. *Bioethics*; 2011; 25: 112–118.

37 V. Berridge. Public or Policy Understanding of History? *Soc Hist Med* 2003; 16: 511–523.

38 Geison, *op. cit.* note 20, p. 32.

39 Lynn Hunt, ed. 1989. *The New Cultural History*. Berkeley: University of California Press.

40 K. Jenkins. The End of the Affair: On the Irretrievable Breakdown of History and Ethics. *Rethinking History* 2007; 11: 275–285, p. 283.

41 Ibid: 275.

42 Cooter, *op. cit.* note 20, p. 260.

43 Ibid: 259.

44 Ibid.

45 De Vries et al, *op. cit.* note 112, p. 2.

46 Callahan, *op. cit.* note 2, p. 4.

47 A. MacIntyre. 1975. How Virtues Became Vices: Values, Medicine and Social Context. In *Evaluation and Explanation in the Biomedical Sciences*. H.T. Engelhardt & S. Spicker, eds. Dordrecht: Reidel Publishing: 97–111; A. MacIntyre, Why is the Search for the Foundations of Ethics so Frustrating? *Hastings Cent Rep* 1979; 9, no. 4: 16–22.

48 L.B. McCullough. Historical Perspectives on the Ethical Dimensions of the Physician-Patient Relationship: The Medical Ethics of Dr. John Gregory. *Ethics Sci Med* 1978; 5: 47–53, p. 47.

49 Cooter, *op. cit.* note 21, p. 457.

50 McCullough, *op. cit.* note 46, p. 53.

51 R. Gillon. Autonomy and the Principle of Respect for Autonomy. *Brit Med J* 1985; 290: 1806–1808; R. Downie. Is there a Right to be Unhealthy? *Nurs Mirr* 1983; 158: 30; R. Downie. Pervasive Ethical Problems. *Nurs Mirr* 1985; 160: 42.

52 I. Kennedy. 1981. *The Unmasking of Medicine.* London: Allen and Unwin: 2.

53 R. Porter. The Patient's View: Doing Medical History from Below. *Theory and Society* 1985; 14: 175–198.

54 M. Warnock. 1985. *A Matter of Life: the Warnock Report on Human Fertilization and Embryology.* London: Basil Blackwell, 1985: 96. For more detail, see D. Wilson. Creating the ‘Ethics Industry’: Mary Warnock, In Vitro Fertilization and the History of Bioethics in Britain. *Biosocieties* 2011; 6: 121–141.

55 M. Warnock. 1992. Embryo therapy: The Philosopher's Role in Public Debate. In *Ethics in Reproductive Medicine*. D.R. Bromham, M.E. Dalton & P.J.R. Millican, eds. London: Springer Verlag: 21–31, on p. 31.

56 Wilson, *op. cit*. note 51.

57 O. O'Neill. 2002. *Autonomy and Trust in Bioethics.* Cambridge: Cambridge University Press: 1.

58 Haimes, *op. cit.* note 17, p. 109.

59 Taken from a 1984 newspaper clipping titled ‘Birth of a New Ethic’, held at Archives and Manuscripts, Wellcome Trust Library for the History of Medicine, PP/MLV/C/23/1/6.

60 Cooter, *op. cit.* note 30, p. 1749.

61 Jonsen, *op. cit.* note 29, p. 43.

62 D. Reubi. The Will to Modernize: A Genealogy of Biomedical Research Ethics in Singapore. *Int Pol Soc* 2010; 4: 142–158, p. 144. See also D. Wilson. Who Guards the Guardians? Ian Kennedy, Bioethics and the ‘Ideology of Accountability’ in British Medicine. *Soc Hist Med* forthcoming; Wilson, *op. cit.* note 51; E. Díaz Amado. *Foucauldian Analysis of the Recent History of Bioethics in Latin America*. Presented at 2011 Postgraduate Conference on Bioethics; R. Baker & L.B. McCullough, eds. 2009. *Cambridge World History of Medical Ethics* Cambridge: Cambridge University Press.

63 A. Campbell. Philosophy and Medical Ethics. *J Med Ethics* 1976; 2: 2.

64 B. Culliton & W. Waterfall. Flowering of American Bioethics. *Brit Med J* 1978; 2: 1270–1271, p. 1270.

65 M Warnock. 1984. Scientific Research Must have a Moral Basis. *New Scientist* 15 November: 36.

66 I am not saying here that Warnock and Kennedy supported Conservative ideologies; simply that their arguments for outside involvement mapped onto the government's demand for public accountability and ‘empowered consumers’.

67 I. Kennedy. 1983. *The Unmasking of Medicine*. Second Edition: London: Paladin: 181; M. Warnock. A National Ethics Committee *Brit Med J* 1988; 297: 1626.

68 S. Little. Consumerism in the Doctor-Patient Relationship. *J Med Ethics* 1981; 7: 187–190, p. 187; M. Thomas, ‘Should the Public Decide? *J Med Ethics* 1981; 7: 182–183, p. 183.

69 M. Warnock. Do Human Cells Have Rights? *Bioethics* 1987; 1: 1–14, p. 8.

70 M. Warnock. 2003. *Nature and Morality: Recollections of a Philosopher in Public Life.* London: Continuum: 81. Proceedings and correspondence of the Warnock committee are held at the National Archives, catalogue number FD7/2307.

71 Warnock, *op. cit.* note 51, p. 59.

72 Warnock, *op. cit.* note 65, p. 11.

73 On bioethics as ‘boundary work’, see S. Jasanoff. 2007. *Designs on Nature: Science and Democracy in Europe and the United States*. Princeton: Princeton University Press, 2007.

74 Jonsen, *op. cit.* note 30, p. 46.

75 R. de Vries. How Can We Help? From ‘Sociology in’ to ‘Sociology of’ Bioethics. *J Law Med Ethics* 2003; 23: 279–292.

76 See, for example, J.A. Greene. Making Medicines Essential: The Emergent Centrality of Pharmaceuticals in Global Health. *Biosocieties* 2011; 6: 10–33; D. Kevles. Eugenics, the Genome and Human Rights. *Medicine Studies* 2009; 1: 85–93; J. Maienschein. Regenerative Medicine in Historical Context. *Medicine Studies* 2009; 1: 33–40.

77 Rosenberg, *op. cit.* note 21, p. 41.

78 Ibid: 41.

79 G.J. Agich. The Question of Method in Ethics Consultation. *Am J Bioethics* 2001; 1, no. 4: 31–41, p. 38. See also L. Daston & P. Galison. 2007. *Objectivity.* New York: Zone Books; J. Clifford & G. E. Marcus, eds. 1992. *Writing Culture: The Poetics and Politics of Ethnography*. Berkeley: University of California Press.

80 H. White. The Public Relevance of Historical Studies: A Reply to Dirk Moses. *Hist Theory* 2005; 44: 333–338, p. 337.

81 J.V. Pickstone. 2000. *Ways of Knowing: A New History of Science, Technology and Medicine.* Manchester: Manchester University Press: 220–221.

82 Warnock, *op. cit.* note 65, p. 9.

83 D. Edgerton. In Praise of Luddism. *Nature* 2011; 471: 27–29, p. 28.

84 J. Harris. Sex Selection and Regulated Hatred. *J Med Ethics* 2005; 31: 291–294, p. 291.

85 Josephine Quintavalle, director of Comment on Reproductive Ethics, quoted in Pre-Pregnancy DNA Tests for Genetic Conditions Approved. BBC News online 6 April 2011. Available at: http://www.bbc.co.uk/news/health-12983866. For detail on how the history of eugenics can inform current debates, see Kevles *op. cit.* note 76.

86 M.S. Pernick. 2009. Bioethics and History. In *World History of Medical Ethics.* R. Baker & L.B. McCullough, eds. Cambridge: Cambridge University Press: 16–21, p. 18.

87 J.V. Pickstone. The Rule of Ignorance: A Polemic on Medicine, English Health Service Policy, and History. *Brit Med J* 2011; 342: 633–634.

